# Trajectories of Change in Obesity among Tehranian Families: Multilevel Latent Growth Curve Modeling

**DOI:** 10.1155/2016/2639624

**Published:** 2016-03-03

**Authors:** Mahdi Akbarzadeh, Abbas Moghimbeigi, Hossein Mahjub, Ali Reza Soltanian, Maryam Daneshpour, Nathan Morris

**Affiliations:** ^1^Department of Biostatistics, School of Public Health, Hamadan University of Medical Sciences, P.O. Box 65175-4171, Hamadan, Iran; ^2^Modelling of Noncommunicable Disease Research Center, Department of Biostatistics, School of Public Health, Hamadan University of Medical Sciences, P.O. Box 65175-4171, Hamadan, Iran; ^3^Research Center for Health Sciences and Department of Biostatistics, School of Public Health, Hamadan University of Medical Sciences, P.O. Box 65175-4171, Hamadan, Iran; ^4^Cellular Molecular Endocrine Research Center, Research Institute for Endocrine Sciences, Shahid Beheshti University of Medical Sciences, P.O. Box 19195-4763, Tehran, Iran; ^5^Department of Epidemiology and Biostatistics, Case Western Reserve University, Cleveland, OH, USA

## Abstract

*Objectives*. To evaluate the trajectories of change in obesity within and between Tehranian families, who participated in the Tehran Lipid and Glucose Study (TLGS).* Methods*. This study is a family-based longitudinal design, in four waves. A total of 14761 individuals, within 3980 families, were selected. Three anthropometric measurements, body mass index (BMI), waist circumference (WC), and a body shape index (ABSI), were recorded. Multilevel latent growth curve modeling (MLGCM) approach was used for evaluating the change trajectories in obesity within and between the families.* Results*. The mean age of the subjects in the present study was 33.28 ± 19.01 (range 3–89 years) and 50.1% were male. Obesity was significantly increased (*P* < 0.001). Individuals with more fat become obese slower, whereas families with more fat become obese faster (*P* < 0.001). The initial value and growth rate of WC and ABSI were greater in men than in women, while this result is contrary to BMI (*P* < 0.001).* Conclusions*. Our findings demonstrated that there is an alarming increase in the obesity trend in Tehranian families. The important role of the family in the prevention of obesity is highlighted, underlining the need for public health programs, as family centered educations to lifestyle modification, which can address this emerging crisis.

## 1. Background

Obesity is due to an unbalanced energy equation [1]. Worldwide, obesity is a leading cause of morbidity and mortality, and its prevalence has nearly doubled in the recent years [2, 3]. Burden of obesity disorders, as well as its prevalence and incidence, is increasing in Asia [4]. Many studies have demonstrated a high prevalence and increasing trend for obesity in Iranian people [5, 6]. The role of the parents and the families in obesity prevention is very critical [7]. However, investigating trajectories of change in obesity, over time, is always a major concern for researchers and the previous obesity trend studies, in our country, considered only individual changes to investigate trajectories without considering the families effects.

Body mass index (BMI), defined as weight divided by height squared, is a quantitative measure of obesity. Several measures, as waist circumference (WC) and its derivatives, were developed for amplifying BMI that also consider the body shape. However, the strong correlation between WC and BMI makes it problematic to estimate the excess risks. To overcome this defect, in 2012, N. Y. Krakauer and J. C. Krakauer developed a new measure, “a body shape index” (ABSI), by adjusting for WC with BMI and height, and concluded that ABSI is a better predictor for premature mortality hazard, in the American population sample, than BMI and WC [8]. In the study, obesity was measured by three common indicators, BMI and WC and, a new one, ABSI.

The aim of the study is to assess the shape of trajectories of change in obesity indices, BMI, WC, and ABSI, in Tehranian families that participated in the Tehran lipid and glucose study (TLGS), using multilevel latent growth curve modeling (MLGCM) approach. Specifically, we investigated the longitudinal changes in the measurements, across 12 years, within and between families, relation between initial status and growth rate of the indices within individuals or families, and, also, effects of age and sex on the growth rate of obesity indices.

## 2. Patients and Methods

### 2.1. Study Population

**Figure 1:**
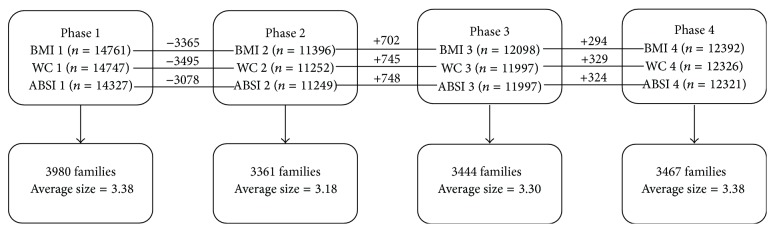
Flow chart of data structure in four phases in Tehran lipid and glucose study. The chart includes time intervals of the study, the number of valid measurements contributing to the analysis in each phase, loss or increase in samples (− or +), the number of families, and average number of members per family. ABSI, a body shape index; BMI, body mass index; WC, waist circumference.

The TLGS is an ongoing longitudinal large-scale community-based study, with a 3-year follow-up period, designed to estimate the prevalence of noncommunicable disorders (NCD) and included a representative sample of residents of 13 districts of Tehran, capital of Iran. The TLGS has been implemented in a multistage stratified (district) cluster (families) random sampling technique, to select more than 15000 people aged >3 years, from March 1999 to December 2001. Phases II, III, and IV were prospective follow-up studies and were performed from 2002 to 2004, 2006 to 2008, and 2009 to 2011, respectively [9, 10]. A total of 14761 individuals (valid case) were selected from the total participating cases in TLGS, including 3980 families, with an average number of 3.38 individuals, among phase I (baseline). At each phase, individuals with at least one valid value for the anthropometric indices entered the analysis. In more detail, the structure of the data is summarized in a flow chart in Figure 1. Informed consent has been obtained from each participant. The TLGS protocol was approved by the Research Institute for Endocrine Sciences, Shahid Beheshti University of Medical Sciences, Tehran, Iran.

### 2.2. Anthropometric Measurements

The anthropometric indices measured included weight (kg), height (m), and waist circumference (cm). Using the World Health Organization standard protocol, WC was measured at the approximate midpoint between the lower margin of the last palpable rib and the top of the iliac crest, by the trained physicians. The BMI was calculated as weight in kilograms divided by height in square meters. The ABSI was calculated as mentioned above.

### 2.3. Statistical Methods

First descriptive data, depicted in Table 1, including mean and standard deviation (SD) of quantitative variables and sex ratio, were reported. To assess the normal distribution of each measurement, among study time points, skewness, kurtosis, and its standard error, as well as Q-Q plot, were used. Also, to compare magnitude of explained variation between and within families of each outcome, we used intraclass correlation coefficient (ICC) at each time point. Then, we conducted a trend analysis, by repeated measures analysis of variance (ANOVA) technique and tested the linear and quadratic trend among four time points.

Since, for individuals of the TLGS data cluster in families, the multilevel analysis was considered and because the outcomes are measured in four time points, data analysis was conducted using MLGCM in three-level framework (time, individual, and family). The MLGCM is a flexible way to analyze latent growth curves, combining structural equation modeling (SEM) and multilevel modeling [11–13]. The MLGCM can incorporate factor analysis and can test variety of causal effects in the SEM framework in longitudinal complex data. These advantages make the model better than ordinary multilevel regression models in longitudinal data. These models were fitted in Mplus version 6.12 (L. Muthén and B. Muthén, Los Angeles, CA, USA) [14].

### 2.4. Model Building Strategy and Assessing the Shape of Changes

**Figure 2:**
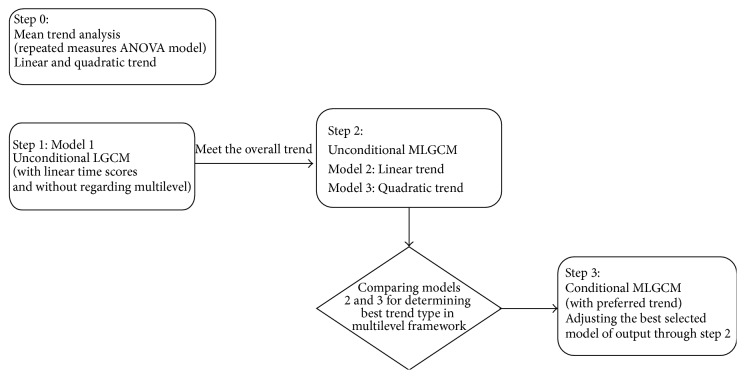
Model strategy building on each anthropometric measurement. Mean trend analysis (step 0); step  1: the conventional latent growth curve was assessed with linear time scores (i.e., 0, 1, 2, and 3 for four phases, resp.); step  2: evaluating shape (linear or quadratic), as a best type of trajectory of changes on the multilevel framework; step  3: conditional multilevel latent growth curve modeling with preferred time scores in step  2, adjusting for age and gender covariates. ANOVA, analysis of variance; MLGCM, multilevel latent growth curve modeling.

For diagnosis of the best shape of changes in the longitudinal multilevel structure data, we performed a strategy in three steps and then the interpretation is according to the preferred model, adjusted for age and sex. The effect of age and sex is considered on the intercept and slope of growth. This plan is shown in Figure 2, as a flow chart.

### 2.5. Model Evaluation Fit Indices

The most common overall fit index in the literature is the Mplus robust maximum likelihood chi-square test that is robust to depart from the normality assumption [15]. Also, Jöreskog suggested using ratio *χ* ^2^/df, as a descriptive index to compare models [16]. To avoid sample size and model complexity dependency problems, the nonnormed fit index (NNFI) and root mean square error of approximation (RMSEA) are commonly used [17, 18]. To evaluate multilevel structural equation model standardized root mean square residual (SRMR) for between model is less likely to detect between-model misspecifications [19]. Suggested rules of thumb exist for interpreting fit indices. For good fit, the cutoffs for the ratio *χ* ^2^/df, RMSEA, SRMR, NNFI, and confirmatory fit index (CFI) are 0–2, 0.05, 0.09, 0.97, and 0.97, respectively, and for acceptable fit, they are 2-3, 0.08, 0.1, 0.95, and 0.95, respectively, [20].

## 3. Results

### 3.1. Descriptive Statistics

The mean age ± SD of the individuals in the present study, at baseline, was 33.28 ± 19.01 (range 3–89 years) and 50.1% were male. Descriptive statistics of age, gender, and anthropometric measurements, for each phase, have been shown in Table 1. Based on repeated measures ANOVA model for assessing polynomial mean contrasts, the linear and quadratic trend were significant (*P* < 0.001). Also, the intraclass correlation coefficients for the three anthropometric measurements are ranged within 0.04–0.13. Also, it is evidenced to consider the family effect of the participant in our data analysis. By taking into account our large sample size, the estimated skewness and kurtosis values, as well as the shape of Q-Q plots, suggested that there is no major departure from the normal distribution of the variables.

### 3.2. Model Building Strategy Output

Results of the three-step strategy, for model building, are summarized in Table 2.

*Step  1 (Model  1): Conventional Linear LGCM.* The first model was used to test whether the anthropometric measurement changed or not during the studied phases. Because the model ignored the two-level structure of the data, the proper model might be unintentionally rejected. For all three anthropometric measurements, the ratio *χ* ^2^/df is very large, RMSEA, CFI, NNFI and SRMR suggesting that this model has acceptable fit to the data, although not good for analysis. This is emerging because individually overall change was assumed in the sequential steps. However, this result is, without regard, multilevel data structure. As shown in Table 1, ICCs of the anthropometric measurements are ranged within 0.07–0.13, 0.05–0.15, and 0.04–0.09 for BMI, WC, and ABSI, respectively.

*Step  2 (Models  2 and 3): Unconditional MLGCM (Linear and Quadratic Shape).* The unconditional MLGCM, in the linear (model 2) and quadratic (model 3) forms, was checked for type of trend shape within multilevel framework. As would be found in Table 2, among three anthropometric measurements, the ratio *χ* ^2^/df is considerably decreased, compared to the conventional LGCM, in step 1 (model 1), while still remaining high. Also, RMSEA, CFI, NNFI, and SRMR suggest that this model has acceptable fit to the data, although not good. Also, in contrast to model 3, model 2 has clearly better fit.

*Step  3 (Model  4): Conditional MLGCM Assessment.* After perusing the two preceding steps to examining trends and shape, in this step, we adjusted the model for the effects of age and gender on intercept and slope of the trajectory. As shown in Table 2, across anthropometric measurements, conditional linear MLGCMs have a better fit than the unconditional linear format.

### 3.3. Final Model Interpretation

The results of the model building strategy preferred the conditional MLGCM, adjusting for age and sex. The model parameter estimations and asymptotic standard errors are represented in Table 3. The effect of age and sex on slop and intercept was significant. The growth rate means are positive, as expected, and significantly different from 0. Among these three means, growth to initial ratio, associated with WC, is greater than others. Variations of initial status in three measurements are significant on individual and family level, as expected. Also, this is true for variations of growth rates of the three measurements, on both individual and family levels, except ABSI on family level. There is a significant correlation between initial status and growth rate, for each of the three measurements, on both individual and family levels, except ABSI on family level. These significant correlations for BMI and WC are negative and positive, for individual and family level, respectively. This indicated that the most obese individuals had a lower rate of increase and families with a high degree of initial obesity had a faster rate of increase. In all three anthropometric measurements, gender effect on initial status and growth rate is significant, while this effect on BMI is positive and negative on WC and ABSI. This means that the initial measurement of BMI and its rate of growth in female subjects are greater than in males, while this is opposite for WC and ABSI. Also, the range of *R*-square of the model for BMI and WC is more than 84%, whereas for ABSI it is less than 65%.

## 4. Discussion

In this study, we used MLGCM adjusted for age and sex on long-term BMI, WC, and ABSI status on TLGS, for investigating obesity trajectories in change of obesity between and within Tehranian families. Our essential findings were (1) obesity among Tehranian people has significantly increased, (2) individuals with more fat become obese slower, while families with more fat become obese faster, and (3) obesity trajectory in men and women is significantly different, while initial value and growth rate of WC and ABSI in men are greater than in women, and, in the meantime, the initial value and growth rate of BMI in men are lower than in women. Since the WC and ABSI are included in body shape, as well as fat, we concluded that, in Tehranian people, men are more obese and have faster growth rates in terms of body shape than women. However, in terms of fat, without regarding the body shape, the situation is worse for women than men.

Similar to the present study, several previous reports have shown that obesity is escalating rapidly, in most countries, in the past three decades [2, 3]. However, the studies concerning BMI showed that, in developing countries, obesity level for women is higher than for men, similar to our study. Hosseinpanah et al. assessed obesity and abdominal obesity by BMI, WC, and waist-to-hip ratio, based on the 3 first phases of TLGS. They used a logistic model for each phase and demonstrated an increasing in prevalence of obesity and abdominal obesity in Tehranian people [6]. Mirzazadeh et al. investigated obesity through a meta-analysis in Iran, based on 58 studies, and demonstrated that obesity prevalence in women is greater than in men and rates increase with age, especially in women [21].

Our study differs from other studies in two ways. First, all the studies investigate obesity measurements, without considering family effect. Our study found obesity trajectories among families by MLGCM in a wide age interval by adjusting the effects on intercept and slope of the trajectory. Also the age effect could be adjusted by stratification in a new model as Multigroup Analysis (MGA) approach.

Second, according to our knowledge, because ABSI has been introduced recently, there is no study to investigate the obesity trajectory by the indicator.

In longitudinal studies, encounter with missing data is unavoidable. We used full information maximum likelihood approach to conduct missing at random mechanism that is more efficient and has less bias, compared to common methods, such as mean imputation, listwise and pairwise deletion.

In summary, the present study, despite significant differences between families, suggested that the obesity trend among the Tehranian people and their families is alarming and, therefore, the burden of obesity in the near future will be problematic.

Therefore, there is a need for public health interventions and approaches to lifestyle modification, which can address this emerging crisis, and we believe that these public health programs should be more family centered.

## Figures and Tables

**Table 1:** Statistical description of observed anthropometric variables.

Variables	*N* (valid)	Mean (SD)	Skewness (SE)	Kurtosis (SE)	ICC
BMI (kg/m^2^)					
Phase 1	14761	24.311 (5.94)	0.01 (0.02)	−0.25 (0.04)	0.069
Phase 2	11396	25.477 (5.78)	0.14 (0.02)	0.20 (0.04)	0.114
Phase 3	12098	25.901 (5.70)	0.38 (0.02)	0.76 (0.04)	0.132
Phase 4	12392	26.648 (5.61)	0.55 (0.02)	0.97 (0.04)	0.058
WC (cm)					
Phase 1	14747	81.303 (16.02)	−0.25 (0.02)	−0.42 (0.04)	0.055
Phase 2	11252	86.173 (15.75)	−0.19 (0.02)	−0.27 (0.04)	0.077
Phase 3	11997	86.705 (15.81)	−0.11 (0.02)	−0.28 (0.04)	0.099
Phase 4	12326	90.820 (14.77)	0.13 (0.02)	0.32 (0.04)	0.149
ABSI					
Phase 1	14327	0.771 (0.05)	0.39 (0.02)	2.66 (0.04)	0.082
Phase 2	11249	0.788 (0.05)	−0.11 (0.02)	1.20 (0.04)	0.038
Phase 3	11997	0.783 (0.06)	−0.24 (0.02)	0.33 (0.04)	0.047
Phase 4	12321	0.804 (0.05)	−0.58 (0.02)	6.58 (0.04)	0.085
Age (year)	15010	33.282 (19.00)	0.05 (0.01)	−1.00 (0.03)	
Sex (Male/female)	0.72				

ABSI, a body shape index; BMI, body mass index; ICC, intraclass correlation; SD, standard deviation; WC, waist circumference.

**Table 2:** Model fit indices in the model building process for body mass index, waist circumference, and a body shape index.

Index	Strategy step	Model	*χ* ^2^ (df)^*∗*^	RMSEA	CFI	NNFI	SRMR	SRMR
							(Within)	(Between)
BMI (kg/m^2^)	Step 1	Model 1	624.034 (5)	0.08	0.989	0.987	0.021	—
	Step 2	Model 2	365.101 (8)	0.048	0.974	0.961	0.004	0.02
		Model 3^*∗∗*^	4908.012 (8)	0.177	0.897	0.821	0.053	0.028
	Step 3	Model 4^*∗∗*^	367.991 (29)	0.043	0.984	0.982	0.005	0.008

WC (cm)	Step 1	Model 1	1569.793 (5)	0.127	0.965	0.958	0.079	—
	Step 2	Model 2	522.656 (8)	0.078	0.962	0.962	0.004	0.042
		Model 3^*∗∗*^	1202.674 (8)	0.088	0.945	0.912	0.009	0.047
	Step 3	Model 4^*∗∗*^	953.432 (29)	0.051	0.975	0.961	0.002	0.023

ABSI	Step 1	Model 1	615.242 (5)	0.079	0.676	0.612	0.236	—
	Step 2	Model 2	266.883 (8)	0.073	0.99	0.834	0.042	0.104
		Model 3^*∗∗*^	989.57 (8)	0.079	0.608	0.563	0.06	0.107
	Step 3	Model 4^*∗∗*^	859.456 (29)	0.048	0.859	0.884	0.011	0.052

Model 1: unconditional LGCM; model 2: unconditional linear MLGCM; model 3: unconditional quadratic MLGCM; model 4: conditional linear MLGCM

^*∗*^All *P* < 0.001

^*∗∗*^Residual variances are equal across time, and, on level 3, residual variances are fixed at zero.

ABSI, a body shape index; BMI, body mass index; CFI, confirmatory fit index; LGCM, latent growth curve modeling; MLGCM, multilevel latent growth curve modeling; NNFI, nonnormed fit index; RMSEA, root mean square error of approximation; SRMR, standardized root mean square residual; WC, waist circumference; *χ* ^2^, chi-square random variable.

**Table 3:** Parameter estimates and asymptotic standard errors of multilevel latent linear growth curve model for anthropometric measurements.

Growth factor	BMI		WC		ABSI	
	Estimate ± SE	*P*	Estimate ± SE	*P*	Estimate ± SE	*P*
Means						
Intercept	22.34 ± 0.19	<0.001	83.93 ± 0.47	<0.001	0.79 ± 0.002	<0.001
Linear	0.85 ± 0.04	<0.001	5.41 ± 0.14	<0.001	0.02 ± 0.001	<0.001
Regression weight						
Sex						
Intercept	1.72 ± 0.11	<0.001	−0.64 ± 0.28	<0.001	−0.014 ± 0.001	<0.001
Linear	0.02 ± 0.02	0.435	−1.20 ± 0.08	<0.001	−0.006 ± 0.001	<0.001
Age						
Intercept	0.64 ± 0.01	<0.001	0.63 ± 0.01	<0.001	0.31 ± 0.01	<0.001
Linear	0.55 ± 0.01	<0.001	0.53 ± 0.01	<0.001	0.47 ± 0.01	<0.001
Individual covariance						
Intercept						
Linear	−0.61 ± 0.07	<0.001	−6.58 ± 0.51	<0.001	0.001 ± 0.001	<0.001
Individual residual variances						
Intercept	16.82 ± 0.48	<0.001	98.63 ± 2.41	<0.001	0.001 ± 0.001	<0.001
Linear	0.49 ± 0.03	<0.001	3.50 ± 0.18	<0.001	0.001 ± 0.001	<0.001
Family variance						
Intercept	2.51 ± 0.32	<0.001	13.12 ± 1.92	<0.001	0.001 ± 0.001	0.006
Linear	0.07 ± 0.01	<0.001	0.65 ± 0.12	<0.001	0.001 ± 0.001	0.329
Family covariance						
Intercept						
Linear	0.23 ± 0.05	<0.001	1.14 ± 0.35	<0.001	0.001 ± 0.001	0.96
Individual *R*-square range						
Phase 1	0.941		0.905		0.573	
Phase 2	0.924		0.884		0.583	
Phase 3	0.921		0.867		0.606	
Phase 4	0.918		0.847		0.583	

ABSI, a body shape index; BMI, body mass index; WC, waist circumference.
